# Role of New Magnetic Resonance Imaging Modalities in Diagnosis of Orbital Masses: A Clinicopathologic Correlation

**DOI:** 10.4103/0974-9233.63077

**Published:** 2010

**Authors:** Nader Roshdy, Maha Shahin, Hanem Kishk, Sherif El-Khouly, Amany Mousa, Iman Elsalekh

**Affiliations:** Mansoura Ophthamic Center, Mansoura University, Mansoura, Egypt; 1 Department of Radiology, Mansoura University, Mansoura, Egypt; 2 Department of Pathology, Mansoura University, Mansoura, Egypt

**Keywords:** Magnetic resonance spectroscopy, Neuroimaging, Orbital masses, Proptosis

## Abstract

**Purpose:**

To evaluate the role of diffusion-weighted magnetic resonance imaging (MRI) and proton magnetic resonance spectroscopy (MRS) in the diagnosis of different orbital masses and their advantages over conventional MRI.

**Materials and Methods:**

The study included 20 patients presenting with proptosis. Every patient was subjected to thorough clinical examination, conventional MRI “T1 weighted, T2 weighted, and postcontrast T1 weighted if needed,” diffusion-weighted MRI, and proton MRS. Orbitotomy was performed, the orbital mass was excised, and histopathological examination was performed.

**Results:**

Diffusion-weighted MRI could differentiate between benign lesions and malignant tumors in 70% of cases; however, overlap occurred in 30% of cases with benign tumors showing restricted diffusion whereas proton MRS could differentiate between benign and malignant tumors in 90% of cases.

**Conclusion:**

Diffusion-weighted MRI and proton MRS can potentially increase the accuracy of diagnosis of orbital masses through *in vivo* tissue characterization. Magnetic resonance spectroscopy seems to be the more accurate modality.

## INTRODUCTION

Proptosis is one of the most common indications for an ophthalmologist to order neuroimaging. The two imaging techniques for the brain and orbit are computed tomography and magnetic resonance imaging (MRI) scans.^1^ It is often unclear whether a cystic lesion is a tumor or an abscess with conventional MRI techniques.^2^ Imaging techniques for visualizing pathology of the brain and orbit continue to evolve and improve. The clinicians now have a wide variety of diagnostic tests from which to choose. Additional noninvasive magnetic resonance characterization of tumors has become available through proton magnetic resonance spectroscopy (MRS) and diffusion-weighted imaging (DWI). Thus, patterns could be used to discriminate different types of tumors. DWI provides image contrast that depends on the molecular motion of water, and it can be easily added to a standard cranial MR examination, with limited increase in time (imaging time ranges from few seconds to 2 min).^3^ While MRI uses the signal from hydrogen protons to form anatomic images, proton MRS uses this information to determine the concentration of brain metabolites *N*-acetyl aspartate, choline, creatinine, and lactate in the tissue examined.^4^ Published work suggests that MRS can reliably differentiate pure tumor and spectroscopically normal tissues.^5^–^7^ However, the exact role of these new techniques is being actively evaluated. The purpose of this study is to investigate the correlation among MRS, DWI, and histopathology in some cases of proptosis.

## MATERIALS AND METHODS

Twenty patients with proptosis were included in this clinical study. They were randomly selected from patients with proptosis attending the outpatient clinic of Mansoura Ophthalmology Center. This study was approved by the Human Subjects Committee of the University of Mansoura, and adhered to the Declaration of Helsinki. Written informed consent was obtained from all participants.

Patients who had bony lesions were excluded from the study. Diagnosis of proptosis was made when patients had readings more than 20 mm using Hertel's exophthalmometer. All patients underwent thorough ophthalmological examination including history taking, assessment of visual acuity and degree and direction of proptosis, measurement of intraocular pressure (IOP), and fundus examination.

Conventional MRI, diffusion-weighted MR imaging (DWI), and MRS of both orbits were done to all patients. Examination was done in MR Unit, Mansoura University Hospitals, using 1.5 T Siemens system equipped with echoplanar capabilities (Symphony).

Diffusion-weighted MR imaging was obtained using a multisection single-shot echo planar imaging sequence (TR/TE/NEX: 2200/139 ms/1) with *b* values of 0, 500, and 1000 s/mm^2^.

The diffusion gradients were applied sequentially in the three orthogonal directions (*X*, *Y*, and *Z*). Sections of 5 mm thickness, interslice gap of 1 mm, FOV of 220-240 mm, and 128 × 256 pixel matrix were used for all images. Scanning time was less than 2 min.

Three types of images were obtained: orthogonal images, trace images, and apparent diffusion coefficient (ADC) maps. The signal intensity of the lesion on DWIs (*b* = 1000) was classified as hypointense (free diffusion) or hyperintense (restricted diffusion).

The ADC maps were calculated automatically by the MRI software. Measurements of ADC were made in different regions of interest (ROI) of the lesions and the mean ADC value was calculated. The ADC values were expressed in 10^−3^ mm^2^/s.

Proton MRS was performed with either chemical shift imaging (CSI) or single voxel spectroscopy (SVS) depending primarily on the appearance of the lesion in the preceding MR images. Four patients underwent SVS examination as they had homogenous, well-defined lesions with no infiltration of surrounding tissues, while the remaining 16 patients underwent CSI.

Water suppression of the dominant water signal by the CHESS technique, outer volume fat suppression as well as magnetic shimming were performed automatically for all patients at the beginning of both SVS and CSI examinations. Curve fitting was performed automatically for all obtained spectra. The criteria for determining the presence of choline in a lesion were the appearance of a clearly identifiable peak at 3.2 ppm spectra. Peak height provided the level of concentration of choline. Any other peaks were not considered as they were not significant in the differentiation between benign and malignant lesions.

Final diagnosis of all orbital masses was made by histopathological examination after orbitotomy. Correlation among the DWI, MRS, and histopathological results of all orbital masses was performed.

## RESULTS

Twenty patients with orbital soft tissue masses were included in this study. They were 10 men and 10 women. Histopathologically, 14 cases had benign lesions whereas the remaining six had malignant lesions. The classification of lesions in the study based on histopathology is presented in Table 1.

**Table 1:** Histopathology of orbital masses

Final histopathological diagnosis	No.
Malignant tumors	
Adenocarcinoma of the lacrimal gland	2
Malignant melanoma	2
Lacrimal gland lymphoma	2
Benign lesions	
Idiopathic orbital inflammatory disease	5
Dysthyroid ophthalmopathy	3
Cavernous hemangioma	1
Lymphangioma	2
Schwannoma	1
Optic nerve glioma	2
Total no.	*20*

On DWIs, 57% of benign lesions appeared hypointense indicating free diffusion. A case of cavernous hemangioma, the two cases of optic nerve glioma, two cases out of five of idiopathic orbital inflammatory disease, and one case out of three of dysthyroid ophthalmopathy appeared hyperintense indicating restricted diffusion. The ADC was high for all benign lesions except for these six cases which had low values. All (100%) malignant tumors appeared hyperintense indicating restricted diffusion and showed low ADC values [Table 2].

**Table 2:** Diffusion weighted imaging of benign and malignant lesions

Tumor	No.	DWI		Apparent diffusion coefficient value (mean) × 10^−3^ mm^2^/S
			
		*b* = 0	*b* = 1000
Benign lesions				
Idiopathic orbital inflammatory disease	5	Hypo	Hypo in three cases	1.71
Dysthyroid ophthalmopathy	3	Hyper	Hypo in two cases	1.93
Cavernous hemangioma	1	Hyper	Hyper	0.72
Lymphangioma	2	Hyper	Hypo	1.95
Schwannoma	1	Hyper	Hypo	2.08
Optic nerve glioma	2	Hypo	Hyper	0.72
Malginant tumors				
Adenocarcinoma of the lacrimal gland	2	Hypo	Hyper	1.09
Malignant melanoma	2	Hypo	Hyper	0.37 → 0.99
Lacrimal gland lymphoma	2	Hypo	Hyper	1.08

DWI, Diffusion-weighted imaging

**Figure 1:**
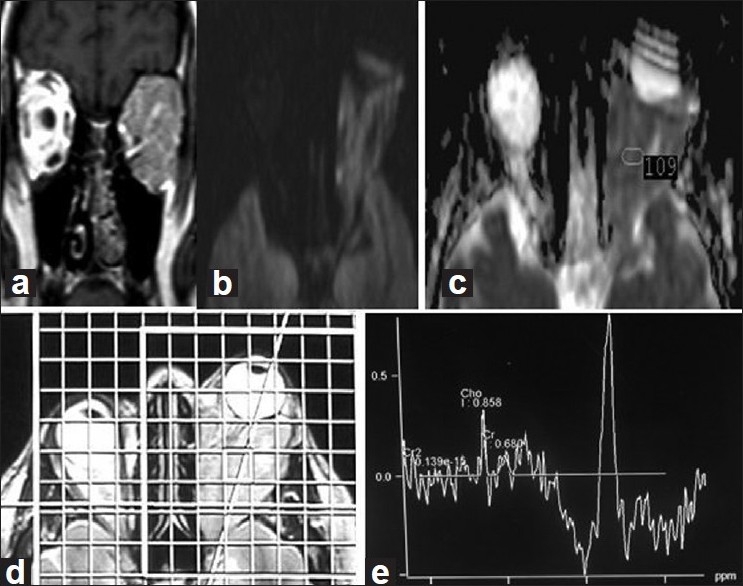
Adenocarcinoma of the lacrimal gland in a 33-year old woman. (a) Coronal T1WI shows large intra- and extraconal mass with infiltration of the extraocular muscles occupying the left orbit. (b and c) Axial DWI and ADC map show hyperintense lesion with low ADC value = 109 × 10^−3^ mm^2^/s indicating restricted diffusion. (d and e) Chemical shift imaging spectroscopy: spectrum shows identified choline peak at 3.2 ppm

**Figure 2:**
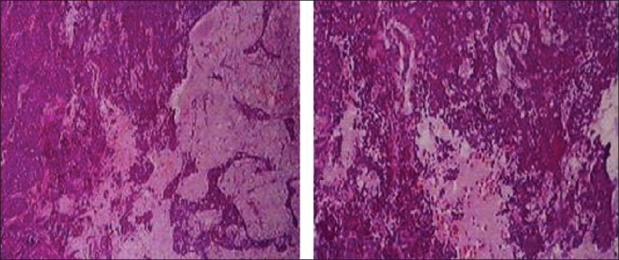
Adenocarcinoma of the left orbit in a 33-year-old woman. The specimen shows pleomorphic, mitotically active cells arranged in sheets and cords, manifest lumen formation and mucin production. There are variable proportions of myxoid and chondroid elements and proliferating epithelial cells that show carcinomatous changes

Diffusion-weighted MRI could differentiate between benign lesions and malignant tumors in 70% of cases. However, overlap occurred in 30% of cases with benign tumors showing restricted diffusion. Figure 1 is a case of adenocarcinoma of the lacrimal gland in a 33-year-old woman. Coronal T1WI shows large intra- and extraconal mass with infiltration of the extraocular muscles occupying the left orbit. Axial DWI and ADC map show hyperintense lesion with low ADC of 1.09 × 10^−3^−^3^mm^2^/s. Histopathology of this case is shown in Figure 2.

**Figure 3:**
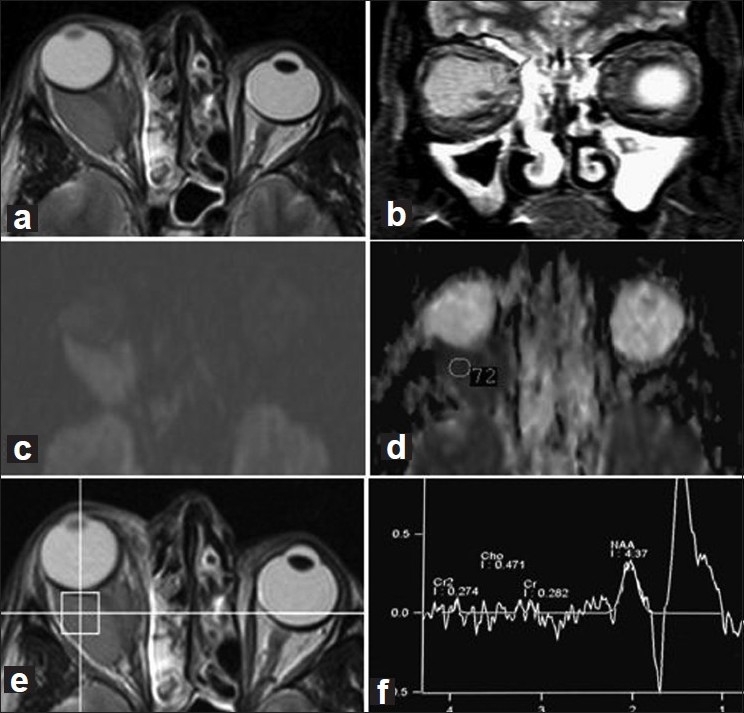
Cavernous hemangioma in the right orbit of a 54-year-old man. (a and b): Axial and coronal fast spin echo show a well-defined homogeneous intraconal mass surrounding the optic nerve which appeared displaced medially and inferiorly (arrow) with subsequent proptosis. (c and d): Axial DWI (*b* = 1000) and ADC map: the lesion is hyperintense on DWI (restricted diffusion), with low ADC value = 0.72 × 10^−3^ mm^2^/s. (e and f) Single voxel spectroscopy shows the absence of choline peak denoting benign nature of the lesion

**Figure 4:**
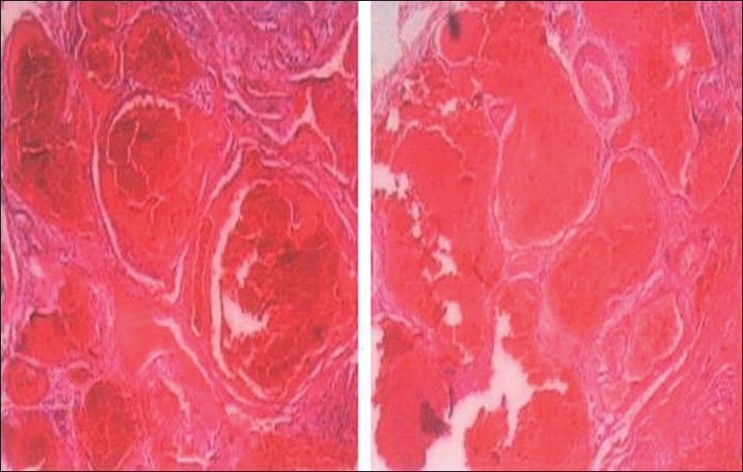
Cavernous hemangioma in the right orbit of a 54-year-old man. The specimen shows large loosely distributed vascular channels associated with orbital hemangioma

In a case of cavernous hemangioma in the right orbit of a 54-year-old man, the axial and coronal fast spin echo show a well-defined homogeneous intraconal mass surrounding the optic nerve which appeared displaced medially and inferiorly with subsequent proptosis. Despite its benign nature, in axial DWI (*b* = 1000) the lesion is hyperintense (restricted diffusion) and ADC map shows a low ADC value of 0.72 × 10^−3^ mm^2^/s Figure 3. Histopathology of this case is shown in Figure 4.

On assessment of MRS, a choline peak was observed in the two cases of optic nerve glioma out of 14 benign lesions (14%), and was present in all malignant tumors (100%) [Table 3]. Chemical shift imaging spectroscopy in a case of adenocarcinoma of the lacrimal gland with a choline peak at 3.2 ppm is presented in Figure 1d and e. Single voxel spectroscopy in a case of a cavernous hemangioma shows the absence of a choline peak denoting benign nature of the lesion [Figures 3e and f]. This case illustrates the false-positive results of DWI which shows restricted diffusion indicating malignancy while MRS shows absent choline peak denoting the benign nature of the lesion.

**Table 3:** Qualitative identification of various Peaks on proton MR spectra of benign and malignant lesions

Tumor	No.	Choline peak	Creatine peak	NAA peak
Malignant tumors				
Adenocarcinoma of the lacrimal gland	2	2+	2+	2+
Malignant melanoma	2	+2	+2	+2
Lacrimal gland lymphoma	2	2+	2−	+1
Benign lesions				
Idiopathic orbital inflammatory disease	5	−5	3	+3
Dysthyroid ophthalmopathy	3	−3	+2	−1
Cavernous hemangioma	1	−	+	−
Lymphangioma	1	−	+	−
Schwannoma	1	−	−	+
Optic nerve glioma	2	+2	+1	+1

− = No identified peak

+ = clearly identified peak

## DISCUSSION

Although abnormalities of diffusion are generally not pathognomonic, diffusion MRI affords information about tissue changes for specific disorders that complement information obtained with standard MR techniques and frequently shows pathology earlier.^8^ Diffusion-weighted imaging is on the basis of the microscopic random (Brownian motion) translational motion of water molecules. Changes in water molecular diffusion can be measured *in vivo* with DWI. This measurement of the self-diffusion coefficient of water indicates the mobility of water within tissue and is called the apparent diffusion. In DWI, a pair of pulsed magnetic field gradients is applied and water molecules that have diffused during the time interval between the applied pulses show a larger signal loss than water with restricted motion. Restricted diffusion appears bright on DWI. Such imaging can be performed without the need for the administration of exogenous contrast medium. It yields quantitative and qualitative information that reflects changes at the cellular level and indicates the integrity of cell membranes.^9^ DWI may be of particular use when contrast material is contraindicated.^10^ Evolving DWI applications include the evaluation of inflammatory, demyelinating, and neoplastic lesions.^1^ It could differentiate between malignant and normal tissues in the liver, kidney, and parotid.^11^–^13^ In cases of neurofibromatosis, the ADC was generally greater than the normal brain.^14^ In this study, DWI correlated with the histopathological diagnosis in 70% of cases of orbital lesions. In the two cases of lacrimal gland lymphoma included in our study, the lesions appeared hyperintense with lower ADC values as compared with the three hyperintense cases of idiopathic orbital inflammation. Similar finding were reported by Kapur *et al*.^15^ in a study to differentiate between the clinically overlapping orbital inflammatory syndrome, orbital lymphoid lesions, and orbital cellulites.^15^ In their study, Kapur *et al.* reported that the brightness of the lesions were higher and the ADC values were lower in the following order: orbital lymphoid lesions; orbital inflammatory syndrome; and orbital cellulites.^15^ This outcome was an important finding as it allows prompt treatment.^15^

Magnetic resonance spectroscopy is based on detecting various proton MR spectra. The four major resonances for MRS are: (1) choline-containing phospholipids, (2) creatine and phosphocreatine, (3) *N*-acetyl aspartate (NAA), and (4) lactate. Reduction of NAA on MRS is a marker of neural loss. Certain tumors have no NAA (e.g., meningioma or metastases) or markedly decrease NAA (e.g., glioblastoma multiforme, and metastasis). Creatine can be increased in hypometabolic state caused by ischemia or tumor (e.g., gliomatosis), or it may be decreased in hypermetabolic states. Choline is a component of cell membranes, and increased choline might suggest increased membrane synthesis in an active proliferating or a solid, hypercellular tumor. Creatine often remains stable in other disease processes and can be used as a control for MRS with levels of other metabolites expressed as a ratio to Cr.^1^ The most widely used clinical application of MRS has been in the evaluation of central nervous system disorders. Although MRS has its limitations and is not always specific, it can be very helpful in the diagnosis of certain entities. For example, a specific pattern of metabolites can be seen in disorders such as creatine deficiency and untreated bacterial brain abscess. It may also be useful in differentiating high-grade from low-grade brain tumors.^4^ It was more sensitive than conventional MRI in grading glioma.^13^ In this study, MRS could differentiate between benign and malignant cases in 90% of cases depending on the presence or absence of the choline peak. False-positive results of both DWI and MRS in cases of glioma of optic nerve may be related to hypercellularity of the lesion.^16^

## CONCLUSION

Advanced MRI techniques, such as MRS and DWI, can provide *in vivo* physiological and metabolic information, complementing morphologic findings from conventional MRI in the clinical setting. Both techniques are noninvasive techniques to monitor changes in the biological structure of tumor tissue. They are a potential diagnostic tool for *in vivo* tissue characterization. The combination of MRS and MRI has led to mapping metabolites from normal and neoplastic tissue within the time limit of a routine study. The applications of these newer techniques are a clinical aid to the ophthalmologist for *in vivo* diagnosis of certain diseases.
